# Vitamin D insufficiency and sleep disturbances in children with ADHD: a case-control study

**DOI:** 10.3389/fpsyt.2025.1546692

**Published:** 2025-03-20

**Authors:** Panpan Zhang, Yang Liu, Yan Ma, Tingting Zhao, Chan Zhang, Hao Sun

**Affiliations:** ^1^ Department of Child Health, Dalian Municipal Women and Children’s Medical Center (Group), Dalian, Liaoning, China; ^2^ Dalian Medical University, Dalian, Liaoning, China; ^3^ Department of Key Laboratory, Dalian Municipal Women and Children’s Medical Center (Group), Dalian, Liaoning, China

**Keywords:** attention deficit hyperactivity disorder, Vitamin D, symptom, children’s sleep habits questionnaire, sleep disordered breathing

## Abstract

**Introduction:**

Attention deficit hyperactivity disorder (ADHD) is a common neurodevelopmental disorders often co-occurring with sleep problems and other physical disorders. Vitamin D regulates sleep and supports normal brain function. Regrettably, no studies have looked at whether vitamin D insufficiency exacerbates sleep problems in ADHD children and further affects ADHD symptoms.

**Objective:**

This study aimed to examine whether vitamin D insufficiency exacerbates sleep problems and ADHD symptoms in children aged 6–14 years.

**Methods:**

This is a case-control study, 260 ADHD children (aged 6-14 years) were enrolled in, of whom 95 had vitamin D insufficiency and 165 had sufficiency. Collected all ADHD symptom severity and functional impairment scales, including Swanson, Nolan and Pelham (SNAP) scale, Integrated Visual and Auditory Continuous Performance Test (IVA-CPT), Conners parents symptom questionnaire (PSQ) and Weiss Functional Impairment Rating Scale-Parent Form (WFIRS-P). All guardians of children with ADHD complete the Children’s Sleep Habits Questionnaire (CSHQ).

**Results:**

The CSHQ total scores of the ADHD children in both groups were significantly higher than 41, which means that ADHD children overall have sleep problems. Compared to ADHD children with vitamin D sufficiency group, we observed significantly higher sleep duration and sleep disordered breathing scores in ADHD children with vitamin D insufficiency group (all *p*< 0.05). However, there was no direct effect of vitamin D insufficiency on the type of ADHD, symptoms or functional impairment (all *p*> 0.05). Further analyses showed a correlation between the CSHQ and symptoms, functional impairment scores in children with ADHD.

**Conclusion:**

Sleep problems are highly prevalent in children with ADHD. Vitamin D insufficiency has a significant impact on both sleep duration and sleep disordered breathing, but no notable direct effects on ADHD symptoms or functional impairment. Our findings underscore the importance of screening for vitamin D insufficiency in children with ADHD, particularly given its association with sleep disturbances, which may indirectly affect symptom severity.

## 1 Introduction

Attention deficit hyperactivity disorder (ADHD), prevalent in neurodevelopment, often emerges in childhood, characterized by persistent, impairing and developmentally inappropriate inattention/disorganization, with symptoms of hyperactivity, impulsivity, and inattention (1–3). Global estimates suggest ADHD prevalence of 5.29% in children and adolescents and more than 65% of ADHD children co-occurring with other developmental, psychiatric and physical disorders (4–6). The precise origin of ADHD remains unidentified, but the dopamine hypothesis gains support from the effectiveness of psychostimulants like methylphenidate, which inhibit neuronal dopamine transporters and prevent dopamine reuptake of from the synaptic cleft, as effective ADHD treatments (7).

A large body of experimental and clinical research indicates that vitamin D plays a role in regulating dopaminergic activity and may be associated with behavioral symptoms in ADHD patients (8, 9). The neuroactive steroid vitamin D has been shown to directly enhance the activity of tyrosine hydroxylase, the key enzyme in dopamine synthesis, by binding to a nuclear receptor known as vitamin D receptors (VDRs) (10). This has implications for the role of vitamin D in neurobiology and could provide insight into potential therapeutic applications. Additionally, ADHD is often accompanied by vitamin D deficiency (11). Studies indicate that vitamin D is essential for the proper functioning of the central nervous system, and may have a role in ADHD (12, 13). Vitamin D supplementation has been shown to positively impact cognitive function across multiple domains, including attention, opposition, hyperactivity, and impulsivity (14).

At the same time, vitamin D plays a role in sleep regulation (15). Specifically, vitamin D insufficiency can exacerbate sleep disorders and is causally linked to sleep difficulties and night awakenings in both children and adults (16–18). Several subjective and objective measures have shown that shorter sleep duration is associated with lower vitamin D levels (19, 20). Some studies have shown that restless leg syndrome is more common and severe in vitamin D insufficiency, suggesting that low vitamin D levels have a negative impact on sleep parameters (21).

Sleep problems are common in children with ADHD, with 25-50% experiencing difficulty sleeping, insomnia, night wakings and hypersomnia (22, 23). Adequate sleep is critical for early childhood neurodevelopment (24). Indeed, during early development, sleep plays a key role in healthy cognitive and psychosocial development (25). Numerous researches have highlighted the crucial role of sleep in regulation learning and memory functions (24, 26). Poor quality and reduced quantity of sleep negatively affect the development of cognitive skills and socio-emotional behavior (27).

Regrettably, few studies have investigated the effect of vitamin D insufficiency on ADHD symptoms and sleep. It is unclear whether vitamin D insufficiency accentuates sleep problems in children with ADHD, and whether sleep problems due to vitamin D insufficiency exacerbate ADHD symptoms and function impairments warrants further investigation. We hypothesized that vitamin D insufficiency would independently contribute to poorer sleep quality and increased functional impairment in children with ADHD. Therefore, we conducted this study to investigate the effect of vitamin D insufficiency on sleep and symptoms in children with ADHD.

## 2 Methods

### 2.1 Study design

This was a case-control study, which was conducted with ethics approval from the Ethics Committee of Dalian Municipal Women and Children’s Medical Center (Group) at the Dalian Medical University in Dalian, China (FEJT-KY-2024-106). The study enrolled subjects from the Department of Child Health at Dalian Municipal Women and Children’s Medical Center (Group) during the period of October 2023 to October 2024. All participants’ parents or guardians provided written agreement, and the research adhered to the ethical guidelines outlined in the Declaration of Helsinki.

### 2.2 Study population

The following is a description of the diagnostic and inclusion/exclusion standards for participants in this clinical study. Per the 5^th^ edition of the Diagnostic and Statistical Manual of Mental Disorders, 5th edition (DSM-5), every participant fulfilled the clinical diagnostic criteria for ADHD (28). The formal diagnosis of ADHD was made by experienced child development and behavior specialist, collected 18-item Swanson, Nolan and Pelham (SNAP-IV) scale, Integrated Visual and Auditory Continuous Performance Test (IVA-CPT), Conners parents symptom questionnaire (PSQ) and Weiss Functional Impairment Rating Scale-Parent Form (WFIRS-P) to assess symptoms and function impairment. The IQ of every child was evaluated using the Wechsler Intelligence Scale for Children-Revised (29). All guardians of children with ADHD complete the Children’s Sleep Habits Questionnaire (CSHQ). All participants were included using consecutive sampling. In order to reduce perception bias, the guardians of ADHD children were strictly screened and required to accompany their children ≥5 days a week before they could fill the forms. Professionals will explain the requirements of filling out the questionnaire to the guardians before they fill it out, and will answer the guardians’ questions at any time during the filling process. This professional is a clinical staff member who is only responsible for the collection of clinical questionnaires and does not participate in the design of the study, compilation and analysis of data, etc.

Inclusion criteria for ADHD children with vitamin D insufficiency were: a) a formal diagnosis of ADHD; b) aged 6 to 14 years; c) 25(OH)D3 ≤ 50nmol/L; Inclusion criteria for vitamin D normal ADHD children were: a) a formal diagnosis of ADHD; b) aged 6 to 14 years; c) 25(OH)D3 > 50nmol/L. All children IQ >70.

Exclusion criteria for all participants were: a) aged <6 years or >14 years; b) having neurological conditions related to central function (e.g. narcolepsy, epilepsy, autism spectrum disorder, intellectual disability) or another significant mental health requiring hospitalization (e.g. depression, anxiety); c) any grave health condition, encompassing conditions like inflammatory bowel disease, cancer history, diabetes, liver or kidney disease; d) ongoing use of any medication, stimulants included; e) incomplete case information.

### 2.3 Study measures

#### 2.3.1 SNAP-IV

Comprising 18 items, the SNAP rating scale features 9 questions about attention deficit disorder (ADD), 5 regarding hyperactivity, and 4 about impulsiveness (30). Hyperactivity disorder (HD) was the term used to describe the collective hyperactivity and impulsivity. Symptom evaluation employs a 4-level Likert scale, spanning from 0 (not at all) to 3 (very much). Scores on the SNAP subscale indicated the degree of ADHD (31).

#### 2.3.2 IVA-CPT

IVA-CPT is a 20-minute examination featuring 500 1s and 2s trails in a pseudorandom order, was used to evaluate sustained selective attention (32). In this examination, the participant clicks the mouse solely upon seeing or hearing a 1, excluding a 2. Trained physicians conducted the tests on every participant, and responses were converted to standardizes scores based on age. This research utilized the full-scale attention quotient (FAQ) and the full-scale response control quotient (FRCQ) to assess the participants’ attention deficit and their ability to control responses, respectively (33).

#### 2.3.3 PSQ

Conners developed this scale to compile an extensive checklist for parents to gather reports on the fundamental issues presented (34). The composition includes 48 elements and 6 subcategories, the composition encompasses indices for character problems index, psychosomatic disorders index, impulsivity index, learning problems index, anxiety index, and hyperactivity index (35). The assessment of the scale utilized a four-tier frequency scale ranging from “never” to “frequently”. An elevated score is associated with the intensity of the symptoms.

#### 2.3.4 WFIRS-P

Comprising 50 questions, the WFIRS-P requires participants’ parents to evaluate their child’s functional disability in the previous month. The version 2 of the WFIRS-P was used in the research. WFIRS-P’s components are evaluated using a four-point Likert-type scale: 0 (never), 1 (sometimes), 2 (often) or 3 (very often), cumulatively yielding six distinct domain scores (Family, Life Skills, Social Activities, Child’s Self- Concept, Learning and School and Risky Activities). Elevated scores in every WFIRS- P domain index was indicative of greater functional impairment (36).

#### 2.3.5 CSHQ

The CSHQ consists of a 35-item parent questionnaire. Parents are need to recall their children’s sleeping behaviors over a “typical” recent month. Items are assessed on a tripartite scale: “usually” indicates happening 5-7 times weekly; “sometimes” denotes 2-4 times weekly; and “rarely” represents 0-1 times weekly. Certain item scores will be inverted and require conversion for subsequent computations. Conceptually, the CSHQ is segmented into eight distinct subscales, each representing different sleep aspects: Bedtime Resistance, Sleep Anxiety, Sleep Onset Delay, Parasomnias, Sleep Duration, Sleep Disordered Breathing, Daytime Sleepiness, Night Wakings (37). The sum of the scores across the eight dimensions comprises the CSHQ total score, which reflects the overall quality of sleep. Higher scores indicate poorer sleep quality, CSHQ total score above 41 points was considered indicative of poor sleep quality.

#### 2.3.6 Vitamin D testing

Serum 25(OH)D3 testing using AB SCIEX liquid chromatography- tandem mass spectrometry (HPLC MS TRIPLE QUAD 4500MD). Global Consensus Recommendations on Prevention and Management and Academy of Medicine, recommends the following classification of serum vitamin D status: deficiency (<30 nmol/L), insufficiency (30–50 nmol/L), sufficiency (>50 nmol/L) (38, 39). Besides, the Institute of Medicine issued a report stating that 25-hydroxyvitamin D concentrations of 50 nmol/L are adequate (40). Based on the above recommendations, in this study, we used 25(OH)D3≤ 50nmol/L to represent vitamin D insufficiency and 25(OH)D3> 50nmol/L to represent vitamin D sufficiency.

#### 2.4 Statistical analyses

Analysis of the data was conducted with the SPSS software, version 26.0. The presentation of continuous variables was in the form of Mean ± SD. Student’s-t test for normally distributed, while the Mann-Whitney U test for the non-normally date. Categorical data are expressed in amount (%). The chi-square or corrected chi-Square test were used to analyzed the difference between categorical data. Spearman to analyze the clinical characteristics and CSHQ scores. *p*< 0.05 was deemed statistically significant.

## 3 Results

### 3.1 Characteristics of participants

This study encompassed 260 children aged between 6 and 14 years diagnosed with ADHD, of whom 95 had vitamin D insufficiency (39.93± 6.89 nmol/L) and 165 had sufficiency (66.71 ± 14.39 nmol/L). The study’s clinical demographic characteristics are listed in **Table 1**. Basic characteristics like age, males, Body Mass Index (BMI), maternal smoking and drinking during pregnancy, birth history, feeding history and electronic screen exposure, exhibited no statistical difference between the groups (all *p*>0.05).

**Table 1:** Characteristics of ADHD participants.

	ADHD+ vitamin D insufficiency group	ADHD+ vitamin D sufficiency group	Statistics
Subject (n)	95	165	
25 (OH)D3 (nmol/L), means (SD)	39.93 ± 6.89	66.71 ± 14.39	
Age (years), means (SD)	8.43 ± 2.02	8.08 ± 1.73	*Z*=-1.263, *p*=0.207
Male n (%)	74 (77.89)	122 (73.94)	*χ*²=0.508, *p=*0.476
BMI (kg/m^2^), means (SD)	17.48 ± 3.29	17.50 ± 3.73	*Z*=-0.251, *p*=0.802
Maternal smoking during pregnancy n (%)	1(1.05)	1 (0.61)	*χ*²=0.000, *p=*1.000
Maternal drinking during pregnancy n(%)	0(0)	0(0)	_
Term birth n(%)			
< 37 w	8 (8.42)	9 (5.45)	*χ*²=4.708, *p=*0.095
37- 42 w	81(85.26)	153 (92.73)	
>42 w	6 (6.32)	3 (1.82)	
Cesarean n (%)	47 (49.47)	70 (42.42)	*χ*²=1.211, *p=*0.271
Breastfeeding for at least 6 months n(%)			
Breast feeding	40 (42.11)	91 (55.15)	*χ*²=4.914, *p=*0.086
Artificial feeding	18 (18.95)	19 (11.52)	
Mixed feeding	37 (38.95)	55 (33.33)	
Balanced diet n(%)	23 (24.21)	50 (30.30)	*χ*²=1.108, *p=*0.292
Electronic screen exposure>1 h/day n(%)	28 (29.47)	38 (23.03)	*χ*²=1.321, *p=*0.250

ADHD, Attention deficit hyperactivity disorder; 25(OH) D3: 25-hydroxyvitamin-D3; BMI, Body Mass Index; Maternal smoking during pregnancy: ≥20 cigarettes per day or every other day; Maternal drinking during pregnancy: ≥2g of alcohol per day or every other day.

### 3.2 ADHD types, symptoms and functional scores between groups

The results of the comparison of types, SNAP, IVA-CPT, PSQ and WFIRS-P between the ADHD combined vitamin D insufficiency and sufficiency groups were shown in **Table 2**. No statistical differences were seen between the two groups in the above indicators (all *p*> 0.05).

**Table 2:** ADHD types, symptom and function impairment scores between groups.

	ADHD+ vitamin D insufficiency group N=95	ADHD+ vitamin D sufficiency group N=165	Statistics
Types of ADHD n(%)			
Inattention	53	102	
Hyperactive/impulsive	8	12	*χ*²=0.910*, p*=0.634
Combined	34	51	
SNAP, means (SD)			
ADD score	2.02 ± 0.54	1.96 ± 0.53	*Z*=-0.953, *p*=0.340
HD score	1.35 ± 0.70	1.39 ± 0.61	*Z*=-0.428, *p*=0.668
IVA-CPT, means (SD)			
FRCQ	88.17 ± 17.81	88.19 ± 19.51	*T*=-0.007, *p*=0.994
FAQ	65.46 ± 18.81	68.34 ± 20.63	*Z*=-1.025, *p*=0.305
PSQ, means (SD)			
Character problems	0.88 ± 0.53	0.87 ± 0.42	*Z*=-0.196, *p*=0.844
Learning problems	1.86 ± 0.55	1.75 ± 0.53	*Z*=-1.292, *p*=0.196
Psychosomatic disorders	0.38 ± 0.37	0.30 ± 0.36	*Z*=-1.918, *p*=0.055
Impulsivity index	1.39 ± 0.75	1.38 ± 0.71	*Z*=-0.189, *p*=0.850
Anxiety index	0.74 ± 0.54	0.62 ± 0.45	*Z*=-1.745, *p*=0.081
Hyperactivity index	1.33 ± 0.52	1.28 ± 0.48	*Z*=-0.450, *p*=0.652
WFIRS-P, means (SD)			
Family	0.71 ± 0.54	0.81 ± 0.56	*Z*=-1.797, *p*=0.072
Learning and School	0.79 ± 0.47	0.81 ± 0.48	*Z*=-0.216, *p*=0.829
Life Skills	0.99 ± 0.43	0.92 ± 0.39	*Z*=-1.169, *p*=0.242
Child’s Self-Concept	0.86 ± 0.67	0.89 ± 0.72	*Z*=-0.054, *p*=0.957
Social Activities	0.86 ± 0.52	0.76 ± 0.49	*Z*=-1.151, *p*=0.250
Risky Activities	0.30 ± 0.28	0.29 ± 0.21	*Z*=-0.126, *p*=0.900

ADHD, Attention deficit hyperactivity disorder; SNAP, Swanson, Nolan and Pelham scale; ADD, Attention deficit disorder; HD, Hyperactivity disorder; IVA-CPT, Integrated Visual and Auditory Continuous Performance Test; FRCQ, Full-scale response control quotient; FAQ, Full-scale attention quotient; PSQ, Conners parents symptom questionnaire; WFIRS-P, Weiss Functional Impairment Rating Scale-Parent Form.

### 3.3 Sleep problems in ADHD with vitamin D insufficiency

**Figure 1:**
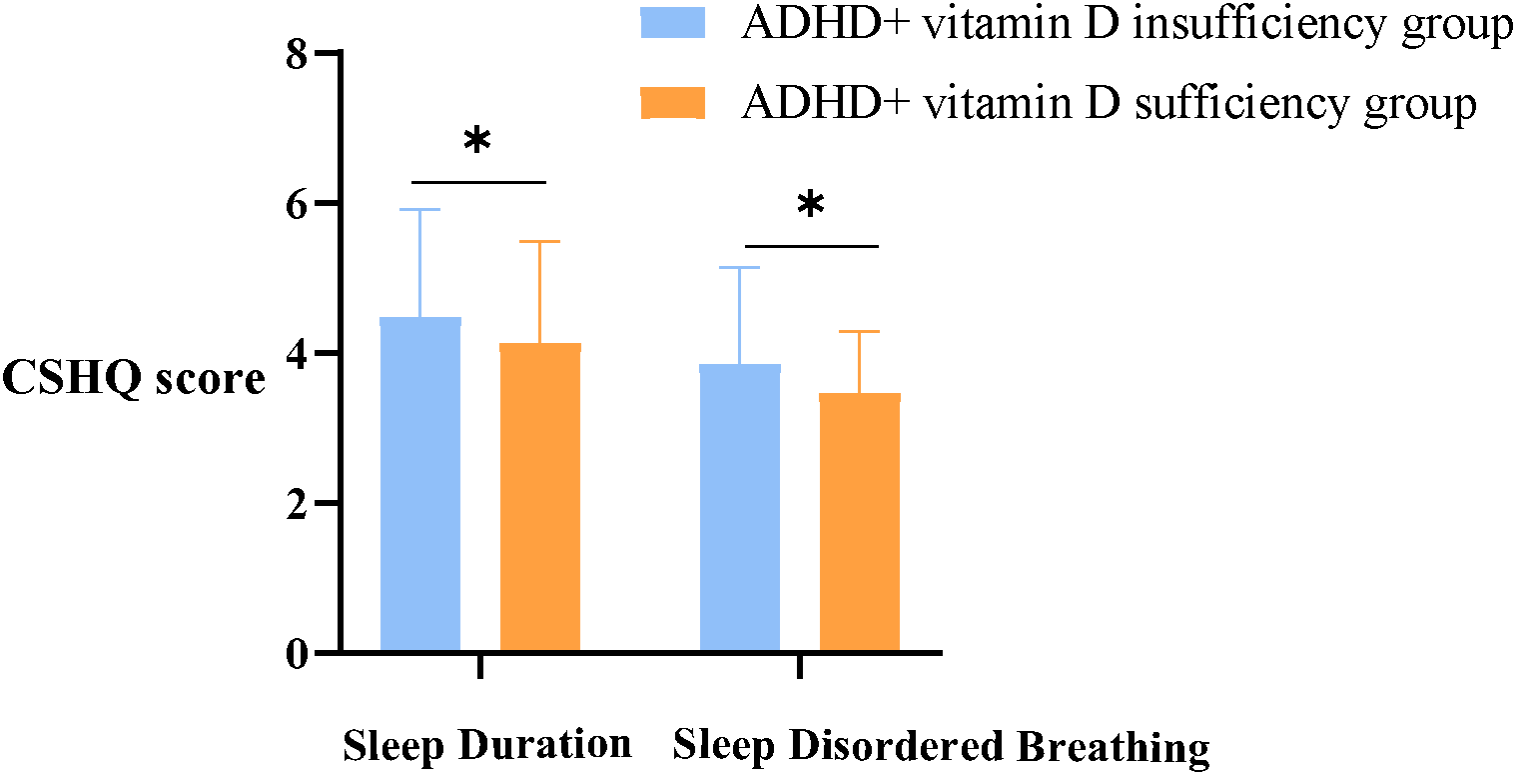
Sleep duration and sleep disordered breathing scores between groups. ADHD, Attention deficit hyperactivity disorder; CSHQ, Children’s Sleep Habits Questionnaire; *, there is a statistical difference between the two groups, *p*< 0.05.

By analysis, it was found that the CSHQ total scores (54.75 ± 7.66) of the ADHD children in both groups were significantly higher than 41, which means that ADHD children overall have sleep problems. About the eight dimensions of CSHQ, the prominent statistically difference between groups were Sleep Duration and Sleep Disordered Breathing scores (all *p*< 0.05) (**Figure 1**), with no statistically significant differences in the remaining dimensions (all *p*> 0.05) (**Table 3**).

**Table 3:** CSHQ scores between groups.

CSHQ means (SD)	ADHD+ vitamin D insufficiency group N=95	ADHD+ vitamin D sufficiency group N=165	Statistics
Hours of sleep per night	9.31 ± 0.74	9.34 ± 0.74	*Z*=-0.175, *p*=0.861
Bedtime Resistance	10.72 ± 3.24	10.67 ± 3.05	*Z*=-0.067, *p*=0.946
Sleep Onset Delay	1.56 ± 0.68	1.50 ± 0.69	*Z*=-0.777, *p*=0.437
Sleep Duration	4.48 ± 1.43	4.13 ± 1.36	*Z*=-2.093, *p*=0.036 *
Sleep Anxiety	7.40 ± 2.33	7.28 ± 2.19	*Z*=-0.388, *p*=0.698
Night Wakings	3.77 ± 1.12	3.72 ± 1.08	*Z*=-0.344, *p*=0.731
Parasomnias	8.86 ± 1.62	8.75 ± 1.71	*Z*=-0.865, *p*=0.387
Sleep Disordered Breathing	3.85 ± 1.29	3.47 ± 0.82	*Z*=-2.438, *p*=0.015 *
Daytime Sleepiness	14.78 ± 2.97	14.69 ± 2.86	*Z*=-0.008, *p*=0.994
CSHQ Total Score	55.06 ± 8.08	54.57 ± 7.42	*T*= 0.500, *p*=0.618

CSHQ, Children’s Sleep Habits Questionnaire; ADHD, Attention deficit hyperactivity disorder; * there is a statistical difference between the two groups, *p*< 0.05.

### 3.4 ADHD symptoms and functional scores are associated with CSHQ

**Figure 2:**
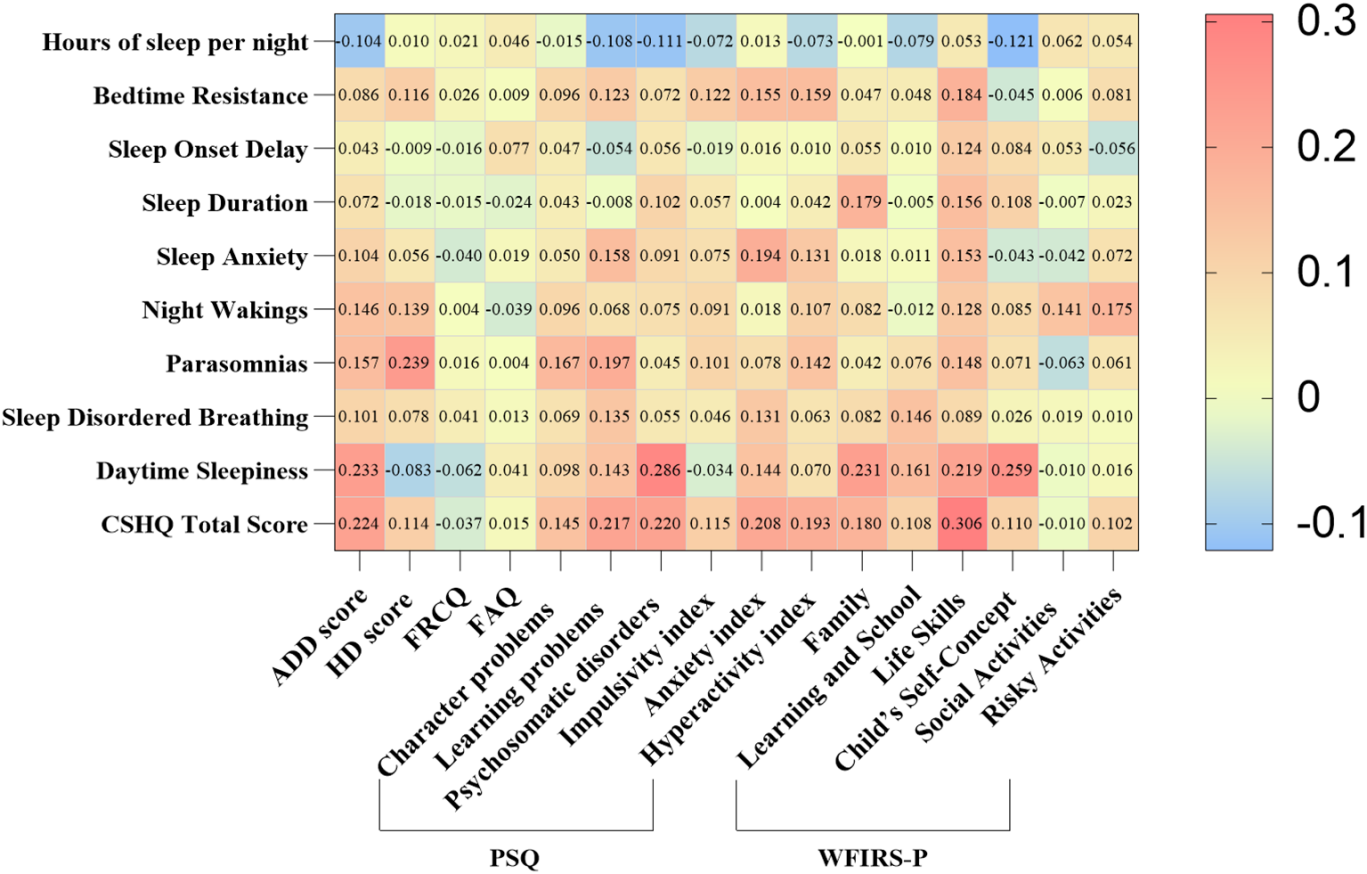
Correlation of symptoms and CSHQ scores in children with ADHD. ADHD, Attention deficit hyperactivity disorder; CSHQ, Children’s Sleep Habits Questionnaire; ADD, Attention deficit disorder; HD, Hyperactivity disorder; FRCQ, Full-scale response control quotient; FAQ, Full-scale attention quotient; PSQ, Conners parents symptom questionnaire; WFIRS-P, Weiss Functional Impairment Rating Scale-Parent Form. Using Spearman to analyze the relationship. Red color represents positive correlation, while blue color represents negative correlation.

We identified that there was a correlation between the CSHQ score and ADHD symptoms, functional impairment scores (**Figure 2**). In particular, Sleep duration was positively correlated with Family and Life skills score in WFIRS-P (r= 0.179, r= 0.157, all *p*< 0.05); Sleep Disordered Breathing were positively correlated with learning problems, Anxiety index in PSQ (r= 0.135, r= 0.131, all *p*< 0.05) and Learning and School in WFIRS-P (r= 0.146, *p*< 0.05). In addition, Hyperactivity index in PSQ were positively correlated with Bedtime Resistance, Sleep Anxiety, Parasomnias and CSHQ total score (r= 0.159, r= 0.131, r= 0.142, r= 0.193, all *p*< 0.05); ADD and HD scores in SNAP were positively correlated with Parasomnias (r= 0.157, r= 0.239, all *p*< 0.05) (**Supplementary Table 1**).

## 4 Discussion

The objective of this study was to examine the impact of serum vitamin D levels on sleep problems, symptoms and functional scores in patients with ADHD. The primary finding was that sleep problems were highly prevalent in children with ADHD, and vitamin D insufficiency primarily influenced sleep duration and sleep disordered breathing, with no discernible direct effect on ADHD symptoms or functional impairment in ADHD children. This is the first study to simultaneously explore the effects of vitamin D insufficiency on sleep and clinical symptoms in children with ADHD.

Sleep problems pose a widespread health issue in children, and ADHD represents one of the significant diseases among the numerous co-morbid sleep disorders seen in this age group. Investigations have showed that the occurrence of ADHD in children alongside other sleep disorders is 74.6%, significantly higher than the previously reported range of 4.2%-6.5% in China (41, 42). Meanwhile, inattention and hyperactivity/impulsivity of ADHD were associated with sleep problem severity (43). This is consistent with our findings that CSHQ total scores in children with ADHD were generally higher than 41 and that CSHQ subscales correlated with ADHD symptom and functional impairment scores. Sleep problem may be a characteristic feature of ADHD or may interact with the disorder’s symptoms, either worsening or being worsened by them. Although it remains challenging to establish a causal relationship between ADHD and sleep problems, the clinical assessment of sleep in ADHD children is critically important.

While vitamin D’s main role is to maintain bone balance, its association with various other health issues like allergy, cardiovascular disease, infectious diseases, glucose metabolism/insulin resistance, etc (17, 44). Lately, an increasing body of research has revealed a connection between vitamin D and sleep problem. It has been observed that insufficient vitamin D levels correlate with reduced sleep duration, and sufficient vitamin D seems essential for sustaining sleep and minimizing night awakenings (15). Our study also found significant alterations in sleep duration in ADHD children with vitamin D insufficiency. Shortened sleep duration is a contributing factor to increased daytime sleepiness, inattention, and oppositional defiant disorder symptoms in children (45). While the precise process through which vitamin D influences sleep remains ambiguous, the crucial factor in this connection appears to be the manifestation of VDRs in brainstem regions implicated in sleep (16). Research has revealed the presence of VDRs in brain regions implicated in sleep control, including the prefrontal cortex, cingulate gyrus, hippocampal dentate gyrus, caudate nucleus, and the substantia nigra; indicating vitamin D’s involvement in sleep control (18, 46).

Our study found that vitamin D insufficiency had a significant effect on sleep disordered breathing in children with ADHD. Children with sleep disordered breathing often have difficulty paying attention, focusing, and being hyperactive during the day. Sleep disordered breathing can also impair memory and executive function, which are important for managing ADHD symptoms (51). Reduced levels of vitamin D in the serum are linked to a heightened risk of respiratory infections and a greater occurrence of allergic rhinitis (52, 53). Recurrent respiratory infections and immune system imbalance can result in tonsillar hypertrophy and chronic rhinitis. Both conditions increase the risk of sleep disordered breathing, particularly obstructive sleep apnea (OSA). OSA is a relatively common disorder in children, impact 3% to 4% of the total child population and is characterized as a disease with low inflammation. Two research showed a linear relationship between the levels of vitamin D and the likelihood of developing OSA (54). Furthermore, vitamin D could inhibit T cell growth, promote Foxp3^+^ Treg cells activation, and reduce Th17 cell differentiation and transcription, thus diminishing allergic inflammation (55, 56). This may be a possible mechanism for the occurrence of sleep disordered breathing in ADHD children with inadequate vitamin D levels.

In addition, serum vitamin D was negatively correlated with several biomarkers of systemic inflammation, such as high-sensitive C-reactive protein (hs-CRP), IL-6, and TNF-α levels (47). Studies have shown that increased levels of hs-CRP and IL-6 are associated with sleep disturbances, suggesting that inflammation can disrupt normal sleep patterns (48). And less than 5.5 hours sleep duration was significantly linked to increased hs-CRP levels (49). This means that the relationship between sleep and the immune system is bidirectional: lack of sleep can impair immune function, while an unbalanced immune response can disrupt sleep. Inflammatory mediators not only affect sleep, but also have an important impact on ADHD. It has been reported that IL-6 levels are significantly higher in patients with ADHD compared to their peers without ADHD (50). Elevated IL-6 may contribute to neuroinflammatory processes that affect brain function and behavior in ADHD patients. However, the effects of systemic inflammation caused by vitamin D insufficiency on sleep and ADHD need further exploration.

Despite the effects of vitamin D insufficiency on sleep in children with ADHD and may further contributary effect of sleep disturbances on ADHD symptoms, we did not identify any significant differences in clinical symptoms or functional impairment between children with ADHD who were vitamin D insufficient and those with normal vitamin D levels. Recent years have seen several studies investigate the relationship between vitamin D status and ADHD in children and adolescents; however, the results have been inconsistent (9, 13). One study found that improvement in attention was associated with an increase in 25(OH)D3 after one month of vitamin D supplementation in children with ADHD (57). A randomized controlled trial showed that while vitamin D and magnesium supplementation improved cognitive functions in children with ADHD, it did not significantly affect hyperactivity scores or conduct problems (58). A systematic review indicates that lower serum vitamin D levels are associated with ADHD, but the overall effect sizes are small (9). The contradictory outcomes described above may be related to the emergence of confounding factors or may be due to differences in sample sizes, demographic characteristics, and other features included in the study design. Our study showed that vitamin D insufficiency had no direct effect on ADHD symptoms. But given the direct effect of vitamin D insufficiency on sleep and the clear correlation between sleep and ADHD symptoms, we believe that vitamin D insufficiency has a potential effect on ADHD symptoms, which requires further exploration of the potential association between the two by expanding the sample size and using polysomnography.

Sleep plays an important role in neurodevelopment. Although this study found that vitamin D insufficiency did not have a direct effect on ADHD symptoms, its direct effect on sleep was definitive, and there is a correlation between sleep and ADHD symptoms. This alerts clinicians to the importance of assessing children’s sleep in the management of ADHD and to further explore possible causes of sleep problems in children, especially vitamin D levels. Further guide parents to improve the sleep of children with ADHD by supplementing vitamin D, lifestyle modifications, etc. At the same time, there is value in focusing on the effects of inadequate vitamin D levels early in life on sleep and neurodevelopment later in life. It is well known that vitamin D insufficiency in infancy and early childhood excites the sympathetic nerves, and children often exhibit irritability and sleep problems. However, few studies have explored the neurodevelopmental impact of sleep problems caused by vitamin D insufficiency in infancy and early childhood. And this has important implications for the future prevention and treatment of neurodevelopmental disorders in children, especially ADHD.

This study has some limitations. Firstly, sleep information was mainly obtained from subjective parental questionnaires. Subjective methods offer various advantages, including non-invasive collection and low cost. However, the use of subjective sleep measures is considered be affected by expectations, psychological influences, and responder bias. Polysomnography is widely recognized as the “gold standard” for measuring sleep, collecting comprehensive physiological data over full night, including brain activity, eye movement, heart rate, and blood oxygen levels (59). There were discrepancies between subjective report sleep and objective sleep measures. Therefore, there is a need to use a combination of subjective and objective to better understand sleep in children with ADHD. Secondly, the children with ADHD included in this study were all from northeastern China to ensure data stability and reduce the influence of latitude and longitude on the data, representing ADHD children in the north. However, the selected children are still geographically limited, and further expansion of the geographic area and sample size is needed to represent a wider range of children with ADHD. Thirdly, all participants in this study were ADHD children, and there was no investigation of vitamin D and sleep in non-ADHD children. In subsequent studies, we will further recruit non-ADHD children to observe whether there is a difference in the effect of vitamin D deficiency on sleep between the two groups of children. Lastly, longitudinal studies could better explore the effect of comorbid vitamin D insufficiency on ADHD symptoms and sleep, and in the next step we will conduct a prospective cohort study further explore the causal relationship.

## 5 Conclusion

Our research revealed that sleep problems are highly prevalent in children with ADHD and that vitamin D insufficiency further exacerbates sleep problems in children with ADHD, especially sleep duration and sleep disordered breathing. Nevertheless, vitamin D insufficiency does not appear to direct affect ADHD symptoms and functional impairment.

## Data Availability

The original contributions presented in the study are included in the article/**Supplementary Material**. Further inquiries can be directed to the corresponding author/s.
