# Supplementary material for: Vitamin D insufficiency and sleep disturbances in children with ADHD: a case-control study
Source: Front Psychiatry. 2025 Mar 20;16:1546692. doi: 10.3389/fpsyt.2025.1546692 (PMC11966964; doi:10.3389/fpsyt.2025.1546692)
Supplement: Supplementary file 1 [file Table1.docx]

| CSHQ |  | **SNAP** | | **IVA-CPT** | | **PSQ** | | | | | | **WFIRS-P** | | | | | |
| --- | --- | --- | --- | --- | --- | --- | --- | --- | --- | --- | --- | --- | --- | --- | --- | --- | --- |
|  |  | ADD | HD | FRCQ | FAQ | Character problems | Learning problems | Psychosomatic disorders | Impulsivity index | Anxiety index | Hyperactivity index | Family | Learning and School | Life  Skills | Child’s Self-Concept | Social Activities | Risky Activities |
| Hours of sleep per night | r | -0.104 | 0.010 | 0.021 | 0.046 | -0.015 | -0.108 | -0.111 | -0.072 | 0.013 | -0.073 | -0.001 | -0.079 | 0.053 | -0.121 | 0.062 | 0.054 |
| Bedtime Resistance | r | 0.086 | 0.116 | 0.026 | 0.009 | 0.096 | 0.123* | 0.072 | 0.122* | 0.155* | 0.159* | 0.047 | 0.048 | 0.184** | -0.045 | 0.006 | 0.081 |
| Sleep Anxiety | r | 0.043 | -0.009 | -0.016 | 0.077 | 0.047 | -0.054 | 0.056 | -0.019 | 0.016 | 0.010 | 0.055 | 0.010 | 0.124* | 0.084 | 0.053 | -0.056 |
| Sleep Duration | r | 0.072 | -0.018 | -0.015 | -0.024 | 0.043 | -0.008 | 0.102 | 0.057 | 0.004 | 0.042 | 0.179** | -0.005 | 0.156* | 0.108 | -0.007 | 0.023 |
| Sleep Disordered Breathing | r | 0.104 | 0.056 | -0.040 | 0.019 | 0.050 | 0.158* | 0.091 | 0.075 | 0.194** | 0.131* | 0.018 | 0.011 | 0.153* | -0.043 | -0.042 | 0.072 |
| Parasomnias | r | 0.146* | 0.139* | 0.004 | -0.039 | 0.096 | 0.068 | 0.075 | 0.091 | 0.018 | 0.107 | 0.082 | -0.012 | 0.128* | 0.085 | 0.141* | 0.175** |
| Daytime Sleepiness | r | 0.157* | 0.239** | 0.016 | 0.004 | 0.167** | 0.197** | 0.045 | 0.101 | 0.078 | 0.142* | 0.042 | 0.076 | 0.148* | 0.071 | -0.063 | 0.061 |
| Night Wakings | r | 0.101 | 0.078 | 0.041 | 0.013 | 0.069 | 0.135* | 0.055 | 0.046 | 0.131* | 0.063 | 0.082 | 0.146* | 0.089 | 0.026 | 0.019 | 0.010 |
| Sleep Onset Delay | r | 0.233** | -0.083 | -0.062 | 0.041 | 0.098 | 0.143* | 0.286** | -0.034 | 0.144* | 0.070 | 0.231** | 0.161** | 0.219** | .259** | -0.010 | 0.016 |
| CSHQ Total Score | r | 0.224** | 0.114 | -0.037 | 0.015 | 0.145* | 0.217** | 0.220** | 0.115 | 0.208** | 0.193** | 0.180** | 0.108 | 0.306** | 0.110 | -0.010 | 0.102 |

Supplement Table 1. Correlation of symptoms and CSHQ scores in children with ADHD.

ADHD: Attention deficit hyperactivity disorder; CSHQ: Children’s Sleep Habits Questionnaire; SNAP: Swanson, Nolan and Pelham scale; ADD: attention deficit disorder; HD: hyperactivity disorder; IVA-CPT: Integrated Visual and Auditory Continuous Performance Test; FRCQ: full-scale response control quotient; FAQ: full-scale attention quotient; PSQ: Conners parents symptom questionnaire; WFIRS-P: Weiss Functional Impairment Rating Scale-Parent Form. Using Spearman to analyze the relationship. * *p*< 0.05；** *p*< 0.01.
